# Hidden work, frustration and multiple layers of occupational health in emergency situations: a longitudinal study during the COVID-19 pandemic

**DOI:** 10.3389/fpsyg.2023.1042397

**Published:** 2024-01-11

**Authors:** Matej Černe, Darija Aleksić

**Affiliations:** School of Economics and Business, University of Ljubljana, Ljubljana, Slovenia

**Keywords:** hidden work, frustration, occupational health, stress, burnout, well-being, satisfaction with work-life balance, COVID-19

## Abstract

This study examines the effects of the COVID-19 pandemic over time. Specifically, we derive from the organizational model of frustration to propose and test a model of pandemic-induced hidden work affecting employees’ emotional responses of frustration along with behavioral responses and outcomes with respect to occupational health. We thereby develop a concept of multiple layers of occupational health that spans across stress, satisfaction with work–life balance (SWLB), burnout, subjective well-being, and physical health. Based on a longitudinal web-based survey of 198 working professionals conducted at seven points in time (1,143 data points, with 118 fully completed responses across all time points) for a wide range of industries in 2020, the study tests the proposed relationships using random coefficient modeling. The results show that COVID-19-situation-induced hidden work invokes emotional responses of frustration, which in turn influence outcomes referring to multiple layers of occupational health—positively affecting stress and burnout, and negatively influencing SWLB, subjective well-being, and physical health. Implications for research and practice are discussed.

## Introduction

The COVID-19 pandemic fundamentally altered many aspects of working and non-working life. Almost overnight, a ‘new normal’ has emerged characterized by a fear of the unknown, increased stress due to global health risk, and lockdowns. As the entire world was affected by various measurements and restrictions, there was no way for businesses to continue their typical office-based tasks. In order to maintain business operations, organizations were forced to adopt home-office practices, also known as remote working, wherever possible (Angelucci et al., 2020; Brynjolfsson et al., 2020; Hobbs and Hawkins, 2020).^1^ When working from home was not an option (e.g., for essential workers like medical staff, supermarket and production workers), organizations had to find ways to adjust workflows to make them as smooth yet safe as possible.

The pandemic-related situation was uncertain, permeating every aspect of our lives. Employees found the circumstances to be frustrating and were required to be flexible by adapting to different modes and demands of the quickly altering work processes and contexts. At the same time, employees were pushed to be even more competitive and work harder to preserve their individual positions and keep their organizations afloat (Contreras et al., 2020). Amid such uncertainty, employees who were fortunate enough to retain their jobs during the pandemic reported working more and outside of regular hours (Collins et al., 2020; DeFilippis et al., 2020; ILO Monitor, 2020). These pressures prevented individuals from meeting their individual needs, leading to frustration and disengagement (Chong et al., 2020; Serafini et al., 2020; Yu et al., 2023).

While the long-term impacts of the COVID-19 turmoil have yet to become clear, the immediate impacts, such as stress and challenges related to work–life balance due to the changing work hours, can be seen in practice. However, the mechanisms and effects of this situation on health considerations across the span of the (hitherto) two major periods of the pandemic remain virtually unexplored. This absence of insight is important because the short- and long-term effects on multiple dimensions of occupational health (physical, psychological, psycho-social; Burton and WHO, 2010) hold important implications not just for individuals, but their families and communities as well. One should also not overlook the long-term performance of organizations, which vitally depends on the health of their human capital (Slaski and Cartwright, 2002; Ongori and Agolla, 2008).

In an attempt to capture insights related to the ever changing working modes during the pandemic, we narrow in on the aforementioned idea that at times when a national epidemic is declared employees are more likely to work longer and irregular hours (Hill et al., 2003). Such hidden work, i.e., answering work-related phones or emails outside of formal working hours without receiving compensation for that (Fenner and Renn, 2004; Kost et al., 2023), is a relatively understudied phenomenon in the literature, albeit present in practice for a long time. Namely, digitalization has encouraged an “always-on” workplace culture denoted by 24/7 access to information and connectedness with each other anytime, anywhere in the world. Receiving, checking and responding to work-related emails, calls and other messages after office hours has become routine for many employees. During the COVID-19 pandemic, the “always-on” work culture became the *modus operandi*, further blurring the already fuzzy boundaries between work performed within and outside of formal working hours (Penado Abilleira et al., 2021; Aleksić et al., 2023).

Recent research shows that hidden work behaviors are positively related to negative outcomes like family-to-work conflict and decreased perceptions of well-being, while actually contributing positively to work performance ratings (Fenner and Renn, 2010; Eichberger et al., 2021; Kost et al., 2023). Nonetheless, a comprehensive investigation of the ways hidden work affects multiple aspects of occupational health is missing, as is a study of the mechanisms according to which these effects unfold. In so doing, based on prior work on this matter (Petterson and Arnetz, 1998; Mikkelsen et al., 2005; Campbell Quick et al., 2007; Hill et al., 2019) we develop the concept of multiple layers of occupational health that encompass physical, psychological and psycho-social aspects. The presented research is based on the organizational model of frustration (Spector, 1978; Fox and Spector, 1999; Reio, 2011), which describes the effects of environmental sources of frustration on affective responses (i.e., frustration), which in turn affects behavioral responses and outcomes. In line with this model, we treat hidden work as a source of frustration, which stems from a superior source of frustration arising from environmental uncertainty and change (Serafini et al., 2020), where both act as enablers or inhibiters in achieving individual and organizational goals. Specifically, hidden work (i.e., increased workload) and frustration were chosen because the literature suggests that they influence important physical and mental health outcomes (e.g., Fox and Spector, 1999; Whinghter et al., 2008). In a longitudinal study repeated at seven points in time across a seven-time longitudinal study across two periods of the COVID-19 epidemic, the objective of the study was to test the effects of hidden work via the emotional response of frustration on five aspects of occupational health (i.e., stress, satisfaction with work–life balance—SWLB, burnout, subjective well-being, and physical health).

The study attempts to contribute theoretically in three ways, to three distinct yet related fields. First, we aim to contribute to the occupational health literature by conceptualizing and testing the concept of multiple layers of occupational health. Prior research on the matter is scattered across different conceptualizations and considerations of this phenomena. For example, integrative ones include Psychosocial Work Quality and Health (Petterson and Arnetz, 1998) or look at health outcomes from individual (sickness, presentism, absenteeism) and organizational (vigor, vitality, productivity) perspectives (Campbell Quick et al., 2007). In contrast, the majority of occupational health research concentrates on particular dimensions of the concept, like stress, burnout, subjective perceptions of health etc. (e.g., Cooper and Cartwright, 1994; Schaufeli and Greenglass, 2001; Mikkelsen et al., 2005). The presented study attempts to integrate this research and conceptualize a multi-layered concept of occupational health, one that encompasses physical, psychological and psycho-social aspects.

Second, we seek to advance research on work frustration by examining it in the COVID-19 pandemic context. While the original model of work frustration specifies different sources of either mild or severe frustration (Spector, 1978), we complement it by proposing that hidden work arises from environmental sources of frustration due to an uncertain situation, thereby proposing a dual-layered model of sources of frustration. Moreover, we add a novel set of tested outcomes to the model of frustration, i.e., multiple dimensions of occupational health.

Third, we add to research on hidden work. While prior research indicated that hidden work leads to negative outcomes (Barley et al., 2011; Kost et al., 2023), no particular emotional or behavioral responses have been specified, nor consequent health outcomes. We enhance understanding of its effects on occupational health-related outcomes, thereby complementing existing research on hidden work that reveals it contributes to a less favorable work–life balance and perceptions of work overload (Barley et al., 2011; Kost et al., 2023). We advance this stream of research by conceptualizing and testing a mechanism of how hidden work relates to multiple layers of occupational health outcomes by specifically conceptualizing hidden work as a source of frustration.

## Theory and hypotheses

### Hidden work and employee frustration

The COVID-19 pandemic created uncertainty and many work-related challenges that required employees to be adaptable (Bailey and Breslin, 2021). For many, remote working was obligatory rather than being an optional flexible work arrangement, enabling staff to retain their positions and keep contributing to the organization’s goals. Many employees were thrown into an uncertain situation without any clear idea of what the future would bring and how they would need to adapt. In many instances, adapting to the ‘new normal’ was left to them, who frequently experienced greater autonomy and independence (Galanti et al., 2021). Research indicates that having the autonomy to work anywhere at any time increases the frequency of work, and thus work becomes everywhere and all the time (Mazmanian et al., 2013; Chung and van der Horst, 2020). This situation led to increases in hidden work (Angelucci et al., 2020; Brynjolfsson et al., 2020).

Although being able to answer emails anytime and anywhere increases work flexibility, doing so is associated with work overload and stress (Barley et al., 2011). Hidden work has been linked to family-to-work conflict (Kost et al., 2023). Volini et al. (2020, p. 5) argue the pandemic “put more hours into the working day, creating exhaustion and burnout and simultaneously exposing the stress that many workers face in balancing professional and personal demands, as personal commitments and roles (such as being a parent or caregiver) could no longer be separated from work.” Working longer, faster and with an ‘always-on’ work culture, manifested as constantly monitoring work-related information (e.g., via emails and social media), causes feelings of anxiety, insomnia and inefficiency (Salanova et al., 2007; Derks et al., 2015), which we propose acts as a source of frustration.

Workload itself has been empirically demonstrated to act as a source of frustration (Spector et al., 1988; Spector, 1994); the feeling of being upset or annoyed when an instigated goal-response (or expected behavioral sequence) is interrupted or interdicted (Fox and Spector, 1999). For individuals who hold cognitively demanding and autonomous jobs, quantitative workload levels are often high and stem from multiple sources, which frequently can produce negative affective reactions (Whinghter et al., 2008). Indeed, frustration at work can occur when individuals feel disappointed and dissatisfied with factors such as workplace morale, work arrangements (e.g., work hours) and inappropriate use of their resources (Beckman and Simms, 1992; Chang et al., 2014), or when they fall behind on work tasks (Carver, 2004) such as because of the COVID-19-induced situation and the ensuing adaptations required. Indeed, many psychological problems and important consequences in terms of mental health (including stress, anxiety, depression, frustration and uncertainty) during the COVID-19 outbreak emerged gradually (Duan and Zhu, 2020). Studies reported that the spatial distancing, self-isolation, quarantine, social and economic discord, and misinformation (notably on social media) were among the biggest factors contributing to unusual levels of sadness, fear, frustration, feelings of helplessness, loneliness, and nervousness (Ahorsu et al., 2020; Khan et al., 2020; Sakib et al., 2020).

In the organizational model of frustration (Spector, 1978), frustrated events may be understood as situational constraints in the immediate work situation that block individuals from achieving valued work goals or effectively performing (Berkowitz, 1978; Peters and O’Connor, 1980; Lazar et al., 2006). Such constraints may also be viewed as an external environment of the COVID-19 situation and resulting levels of hidden work. They are expected to induce employees’ emotional response of frustration with both the *broader COVID-19-induced situation* and the *work itself*. We therefore propose:

*H1: Hidden work was positively related to frustration during the COVID-19 pandemic.*

### Hidden work, frustration and multiple layers of occupational health

With employees bearing much of the burden of coping with a crisis like the COVID-19 pandemic, creating a healthy and safe workplace for employees should be a top priority for organizations. Still, this seems to be easier said than done. A healthy workplace calls for a holistic approach to health, encompassing physical, psychological and social contributing factors (Kelloway and Day, 2005). According to the WHO (2011), a healthy workplace is one in which workers and managers collaborate to protect and promote the health, safety and well-being of all workers, along with the sustainability of the workplace. It considers four discrete, yet linked, areas (WHO, 2011): (1) health and safety concerns of the physical work environment; (2) health, safety and well-being concerns in the psychosocial work environment; (3) personal health resources in the workplace; and (4) ways of participating in the community to improve the health of workers, their families, and members of the community.

COVID-19 exposed employees to numerous serious threats to their occupational health (Sinclair et al., 2020). These threats included increased physical risk of infection in the workplace, isolation practices (International Federation of Red Cross and Red Crescent Societies, 2020), quarantine and social isolation, disrupted work routines (Javadi et al., 2020), uncertainty (Shanafelt et al., 2020), a weakened immune system due to high stress levels (Segerstrom and Miller, 2004), physical exhaustion, sleep disruption, and fear (Li et al., 2020), as well as feelings of personal danger (Lai et al., 2020). These may be negatively related to multiple areas of what is considered to constitute a healthy workplace.

We have learned from previous crises that employees exposed to unfavorable working conditions are more likely to experience mental health symptoms (Ten Have et al., 2015). Recent studies show that unhealthy workplace conditions related to COVID-19 have already manifested as deteriorating health (Sinclair et al., 2020; Kniffin et al., 2021). Stress, defined as an emotional response depending on an individual’s perspective (Lazarus, 1990; Bonanno and Burton, 2013), is a normal psychological response to an abnormal situation, which COVID-19 certainly is. In turbulent times, stress can bring out unhealthy extremes in people’s attitudes and behaviors (Weinberg and Cooper, 2012). This means it not surprising that employees reported higher levels of hidden work (i.e., workload) and experienced considerable stress during COVID-19 (Stankovska et al., 2020). The relationship between hidden work, frustration and high stress levels can be explained by the health impairment process arising from demands and resources imposed by one’s job (Bakker and Demerouti, 2007). Job demands create a strain for employees, and this strain is exacerbated by a lack of work resources. Uncertainty, financial, public health and job insecurity, together with new risks that require additional activities and efforts to perform a job safely and efficiently (i.e., increased hidden work), lead to escalating job demands. Employees are faced with high workload demands, with little control over their work (Sinclair et al., 2020), causing them to experience feelings of powerlessness and frustration, which in turn can lead to higher levels of stress. The current conditions of frustration require the investing of energy, which can deplete employees’ psychological resource reservoir, thereby increasing work-related stress. We thus propose:

*H2a: Hidden work was positively related to stress via frustration during the COVID-19 pandemic.*

We further propose that hidden work is negatively associated with SWLB via frustration. SWLB is defined as the “overall level of contentment resulting from an assessment of one’s degree of success at meeting work and family role demands” (Valcour, 2007, p. 1512). To assure satisfying experiences in all life domains, individuals must properly allocate their personal resources like energy, time and commitment across several domains (Kirchmeyer, 2000). During the COVID-19 pandemic, many employees experienced an increase in work and family demands, which can drastically exacerbate work–family conflict (Sinclair et al., 2020), which in step increases frustration and decreases SWLB.

According to Valcour (2007), working hours have a negative impact on SWLB. The pandemic increased workloads and caused stress related to balancing work and personal demands (Volini et al., 2020) since employees were often compelled to work extra hours and overtime to complete their tasks (Irawanto et al., 2021). In the early stages of the COVID-19 pandemic, employees were still adjusting to the new setting and, fearful of losing their jobs, may have been doing more hidden work. To lower their stress levels and find solutions to achieve their goals in these challenging times, employees tended to adopt informal and even uncontrollable practices, completing work-related tasks in the private sphere and beginning to engage in work-related thinking and planning in their free time. This often led to frustration, which refers to negative emotions or passive behavior that occur when motivations and needs are not being met because the individual’s goals are hindered (Wang et al., 2016). A recent study found that during COVID-19 employees were often frustrated by the need to prioritize work at the expense of everything else, which decreased their SWLB (Humphries et al., 2020). Employees who perform considerable hidden work are often focused on work-related activities due to the frustration it causes, thus dedicating more time and energy to work activities and hence neglecting social, family and other activities. The literature suggests that individuals who spend too much or too little time in different domains of their lives have a poorer work-life balance (Kuhnle et al., 2012). Thus:

*H2b: Hidden work was negatively related to satisfaction with work–life balance via frustration during the COVID-19 pandemic.*

Building on literature suggesting that adverse working conditions experienced for a longer period cause a strong sense of frustration and resulting occupational burnout (Kuo and Tsai, 2012), we also argue that hidden work is positively related to burnout via frustration. In a stage model of burnout, frustration is placed prior to burnout (Lewandowski, 2003), defined as a prolonged response to chronic emotional and interpersonal stressors on one’s job (Maslach et al., 2001). This phenomenon is made up of emotional exhaustion (i.e., the feeling of being emotionally over-extended and exhausted at work), depersonalization (i.e., the feeling of callousness and cynicism) and professional inefficacy (i.e., a decline in experienced competence and achievement in one’s work; Schaufeli et al., 1993).

The literature suggests that a heavy workload is most directly related to the exhaustion aspect of burnout (Maslach et al., 2001). Too many demands may exhaust employees’ energy to the extent that recovery becomes impossible. Maslach et al. (2001) argue that emotional work is especially draining when an employee’s job requires them to display emotions inconsistent with their feelings. As discussed above, the COVID-19 situation increased workloads and increased job demands of all kinds, including emotional ones (del Carmen Giménez-Espert et al., 2020). Employees accordingly often feel they have too many things to do in a given time period. Recent studies also suggest that emotional demands and workload were among the most prominent psychological risks during COVID-19 (del Carmen Giménez-Espert et al., 2020). Many employees experienced work stressors, threats of job insecurity, feelings of isolation, which together led to emotional exhaustion (Hwang et al., 2020). The uncertainty and constantly changing work environments saw employees perform higher levels of hidden work, resulting in work-related frustration. Preventing employees from coping with work requirements may further affect not only their short-term, but long-term health indicators. Taken together, we propose:

*H2c: Hidden work was positively related to burnout via frustration during the COVID-19 pandemic.*

The COVID-19 isolation measures, uncertainty, online education of children at home, coupled with job insecurity and health and safety concerns have posed unprecedented challenges to maintaining high levels of subjective well-being, defined as an individual’s positive evaluation of life, experiencing pleasurable emotions, high life satisfaction and fulfillment, and a rewarding life (Diener et al., 2002). Recent studies examining the impact of the COVID-19 pandemic on well-being almost universally show that subjective well-being dropped significantly during the pandemic (e.g., Gray et al., 2020; Wanberg et al., 2020; Ruiz et al., 2021). On top of the obvious COVID-19-related reasons for this, we propose that hidden work, followed by frustration, places an additional strain on employees’ perceived well-being. The literature suggests that a high workload is associated with low subjective well-being (Pace et al., 2021) and that the quality of work and non-work life influences overall subjective well-being (Edwards and Rothbard, 1999; Hart, 1999; Erdogan et al., 2012), suggesting the importance of experiencing pleasant emotions in all domains of life. Hidden work is typically a result of time pressure, tight deadlines, and a heavy workload (Ojala, 2011), which were often present in the work context during the COVID-19 pandemic. As mentioned, these unfavorable working conditions produced a strong sense of frustration, i.e., negative emotions resulting from obstacles or disruptions encountered by employees (Fox and Spector, 1999) that, in turn, reduced the quality of their work life. These negative emotions may also spill over from work to non-work life, thereby affecting the quality of non-work life (e.g., spending less time with family, transferring a bad mood and frustration to family members etc.). This leads us to propose:

*H2d: Hidden work was negatively related to subjective well-being via frustration during the COVID-19 pandemic.*

Overwhelming workloads and stress for an extended period of time can bring about emotional and physical exhaustion if individuals fail to improve or cope with their difficult working conditions (Wang et al., 2016). The pandemic caused unprecedented measures to be introduced to curb the virus, which further worsened working conditions. The economic consequences and job insecurity put additional pressure and frustration on employees, who were often willing to work longer hours and sacrifice their physical health just to perform well in those uncertain times, allowing them to retain their jobs and financial security.

**Figure 1:**
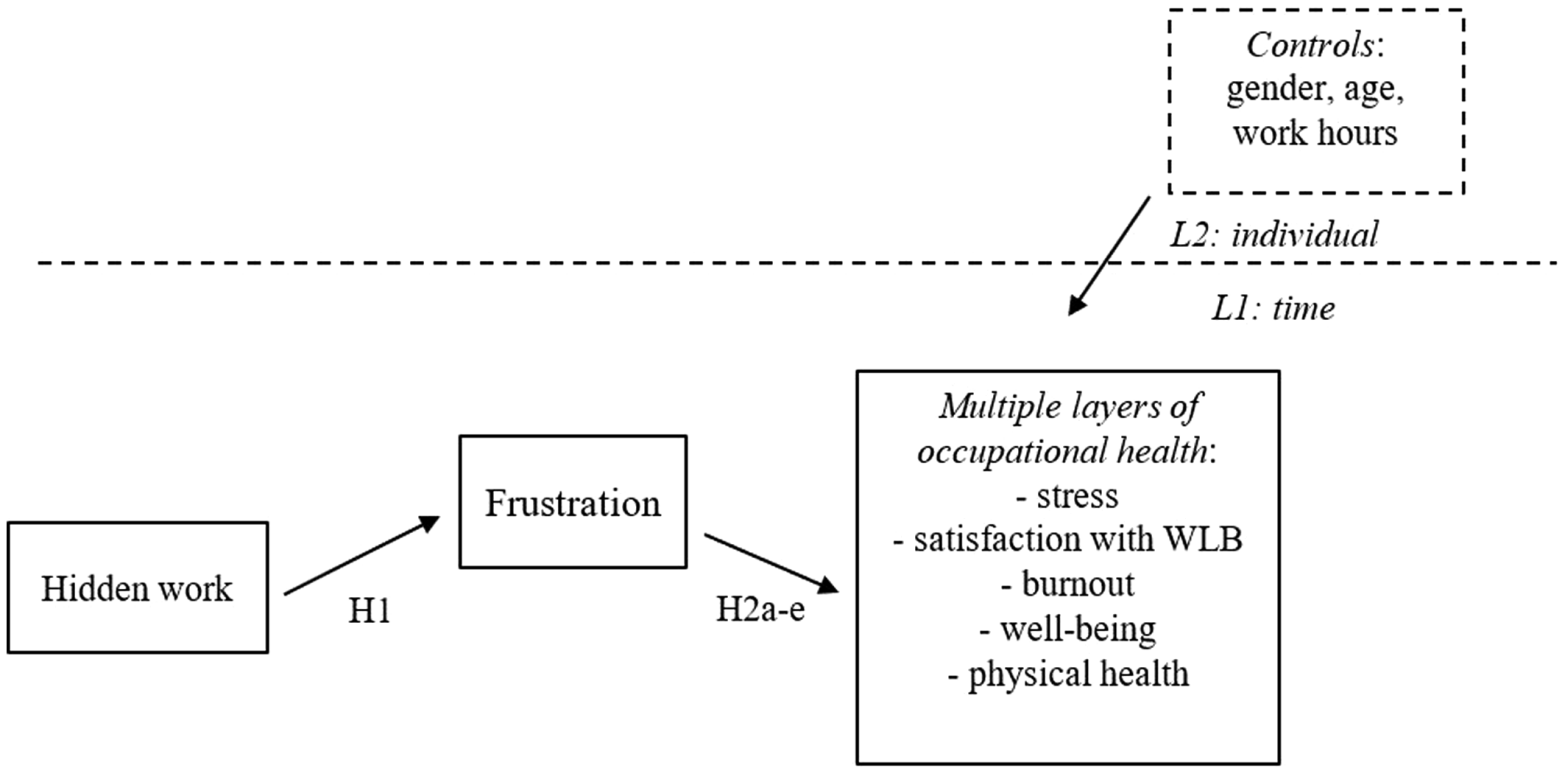
Research model with hypotheses.

Employees’ responses to the stress of perceived job insecurity in the shorter term can be emotional (anxiety, tension, dissatisfaction), psychological (increased heart rate, greater catecholamine secretion) and behavioral (substance use, lack of concentration). In the long term, the accumulation of these responses can bring more permanent adverse mental and physical health consequences (Heaney et al., 1994; Burgard et al., 2009). Namely, workload and time pressures often require constant physical or mental effort and impose potential physical and psychological costs (Sabagh et al., 2022), while job stressors (e.g., job insecurity and work–life conflict) predict the frustration of needs (Sabagh et al., 2022). It is no surprise then that studies show employees were frustrated and highly susceptible to physical exhaustion, depressive symptoms, anxiety, and sleep disorders during the pandemic because of excessive workload and isolation (e.g., Li et al., 2020; Shaukat et al., 2020). Studies also suggest that employees who experience frustration at work, as was common during COVID-19, have lower levels of physical health (De Castro et al., 2010) (see Figure 1). Therefore:

*H2e: Hidden work was negatively related to physical health via frustration during the COVID-19 pandemic.*

Figure 1 summarizes our research model.

## Methods

### Research context

The research was conducted in Slovenia where the first case of the virus was confirmed on 4 March 2020. The growing number of infections saw Slovenia declare an epidemic for the first time on 12 March 2020. Like many other countries around the world, the Slovenian authorities adopted various measures to contain the virus’ spread, such as closing educational institutions, applying restrictions while crossing the border, restrictions on public life, and a ban on all non-essential health service activities.

To limit the economic costs of the COVID-19 pandemic and help employers (and indirectly employees) mitigate the effects of COVID-19, the government adopted the Wage and Contribution Measures Intervention Act (which entered into force on 29 March 2020). This Act regulated the right of employers (and indirectly employees) to apply for a reimbursement of salary compensation paid to employees either unable to work due to business reasons or under the mandatory quarantine. That is, during the time a COVID-19 epidemic was declared in Slovenia, employers could order the absence of workers from work by: (1) arranging work from home due to exceptional circumstances; (2) relying on the use of annual leave by the worker and collective leave; (3) being put on furlough at home (in case of a reduced workload); (4) absence from work to care for children following the closure of kindergartens and schools; and (5) the performance of other work due to the exceptional circumstances. While some enterprises decided to shut down production, many companies switched to remote working, thus causing a boom in remote working across Slovenia. In late April 2020, Slovenia passed the first peak of its COVID-19 epidemic and the government eased its lockdown measures. On 15 May 2020, Slovenia became the first European country to officially declare the end of the COVID-19 epidemic.

Toward the end of summer, the number of COVID-19-infected people began to rise sharply. In October 2020, the situation in Slovenia was much worse than during the first period. On 19 October 2020, a second period of COVID-19 cases occurred in Slovenia, causing the government to again declare an epidemic, activate the National Protection and Rescue Plan, and tighten measures, including a curfew between 21:00 and 06:00. On the same day, primary school students in 6th grade and up together with secondary and university students switched back to distance learning.

As the COVID-19 situation remained serious, in early November the government extended restrictions on movement outside municipalities, extended the distance learning, closed kindergartens, suspended public transport and closed non-essential shops. All gatherings were also banned, except for persons living in the same household. On 16 November 2020, the declaration of an epidemic was formally extended by 30 days. After nearly 2 months of strict restrictions, a temporary relaxation of measures was enacted between 15 December 2020 and 23 December 2020 (i.e., public transport resumed, hair salons, flower shops, car washes and dry cleaners were allowed to reopen). On 22 December 2020, mass antigen testing began in a dozen urban areas across Slovenia, whereas on 27 December 2020, vaccination against COVID-19 started at nursing homes.

### Sample and procedure

We conducted a seven-time [in two periods: Period 1 (before, beginning, peak and end of the first COVID-19 epidemic wave, between February and May 2020) and Period 2 (beginning, peak and softening of the second epidemic wave between October and December 2020)] longitudinal web-based questionnaire study. Table 1 summarizes the COVID-19-related situation in Slovenia in 2020 during each period of the data collection. With the help of a national research agency, using a nationally representative (by age, gender, industry) quota sampling strategy we collected data from 198 employees (who responded to our survey at all seven time points) working in a wide range of industries. Those were sampled from the agency’s registered pool of potential participants who were working professionals. Unique IDs were constructed to ensure the anonymity of participants’ responses and to match responses across the time waves.

**Table 1:** COVID-19-related situation in Slovenia in 2020 during each period of the data collection.

Time	Month	Situation
*Period 1*		
Time 1	Late February	Before the epidemic; preparation of potential measures
Time 2	Mid March	An epidemic officially declared in Slovenia; first phase of the infection spreading; several measures adopted
Time 3	Mid April	peak of the wave-1 epidemic, the most restrictive measures adopted
Time 4	Late May	Official end of the COVID-19 epidemic; lockdown measures gradually eased
*Period 2*		
Time 5	Mid October	An epidemic again declared in Slovenia; infection spreading; several measures adopted
Time 6	Mid November	Peak of the wave-2 epidemic, the most restrictive measures adopted
Time 7	Mid-to-late December	The official epidemic persists, but the lockdown measures gradually and temporarily eased

The sample consisted of 46% of respondents working in public companies, 50% in private companies, with the remaining respondents working in joint ventures. Overall, about 11% of respondents were working in micro-companies with up to 9 employees, 20% in small companies with up to 49 employees, 25% in medium-sized ones with up to 249 employees, and 45% working in large companies with at least 250 employees. The respondents operated mainly in the following industries: education, culture and sport (13%), administration (13%), production (13%), health (10%) and sales (8%). Among the respondents, 42% held a high school diploma and 55% at least an undergraduate diploma.

### Measures

All the focal variables were self-reported and measured on all seven occasions.

*Hidden work* was measured with two items proposed by Kost et al. (2023)—α = 80. Respondents were asked to indicate how often they were working outside their normal working hours without being compensated in the form of extra and/or overtime pay and how often they were responding to work-related emails beyond working hours without being compensated in the form of extra and/or overtime pay. The responses ranged from 1 = never to 5 = very often.

*Frustration* was measured with the following item from Peters et al.’s (1980) scale: “Overall, I experienced very little frustration at work” (reverse scored). The responses ranged from 1 = strongly disagree to 5 = strongly agree.

*Job stress.* Elo et al. (2003) found that the content validity of the single-item measure used for stress symptoms was satisfactory for monitoring stress in different contexts. Job stress was accordingly measured with a single item. We asked the following question: how much stress have you experienced in the last month? A five-point scoring key was used (1 = *I was not under stress at all*, 5 = *I was under extreme stress*).

*Satisfaction with work–life balance (SWLB)*. We measured SWLB using a five-item measure developed by Putrevu and Ratchford (1997). Response options ranged from 1 (“very dissatisfied”) to 7 (“very satisfied”). A sample question is, “In the last month, how satisfied were you with the way you divided your time between work and personal or family life?” (α = 0.96).

*Burnout* was assessed with two items taken from the Maslach Burnout Inventory (MBI) that evaluate emotional exhaustion (i.e., the feeling of being emotionally overextended and exhausted by work) (Maslach et al., 1986). Options for respondents ranged from 1 (“a few times a year or less”) to 6 (“every day”). A value of zero was also added, enabling the respondents to indicate that they had never experienced the described feeling or attitude. A sample item is the following: “I feel tired when I get up in the morning and have the next working day ahead of me” (α = 0.88).

*Subjective well-being* was measured with the following item from Su et al.’s (2016) scale: “Compared to most of my peers, I consider myself more happy.” The responses ranged from 1 = strongly disagree to 5 = strongly agree.

*Self-rated physical health* was measured with an item asking respondents to rate their overall physical health in the past month. Responses ranged from 1 = poor to 5 = excellent.

*Gender*, *age* and *expected average work hours/week* were measured in the first period and incorporated in the model as individual-level control variables.

## Results

### Descriptive statistics

**Figure 2:**
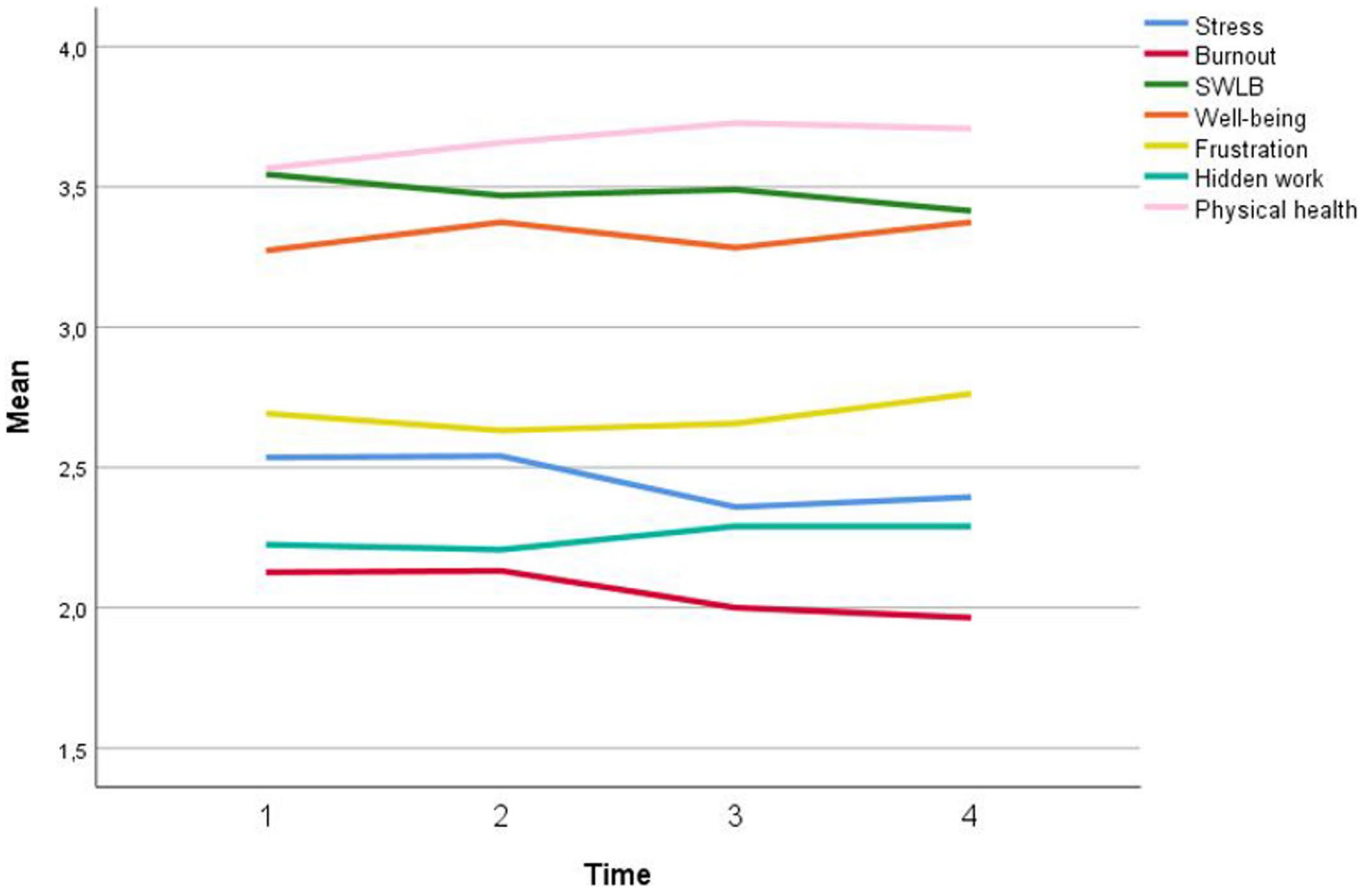
Means of our focal constructs over the seven time points of data collection.

Table 2 shows the means, standard deviations, and correlations of/between the focal variables. Figure 2 portrays the means of our focal constructs over the seven time points of the data collection, across the two periods. As evidenced, frustration (M_period1_ = 2.69; M_period2_ = 3.44; *t* = 9.48; *p <* 0.01), stress (M_period1_ = 2.46; M_period2_ = 3.38; *t* = 13.55; *p <* 0.01) and burnout (M_period1_ = 2.37; M_period2_ = 2.83; *t* = 5.87; *p <* 0.01) significantly increased from Period 1 to Period 2, while other variables exhibit a slight positive trend. In the second period (questionnaire repetitions 5–7), we also collected data on remote work (with 62% of the respondents working remotely at time 6) and various COVID-19-related information, such as care for children at home (50%) or having received state aid benefit (13%).

**Table 2:** Descriptive statistics and correlations.

	Variable	Mean	SD	1	2	3	4	5	6	7	8	9	10
1	Hidden work	2.27	1.18	(0.80)									
2	Frustration	2.92	1.29	0.05	–								
3	Stress	2.74	1.14	0.32^**^	0.15^**^	–							
4	Burnout	2.51	1.25	0.31^**^	0.26^**^	0.64^**^	(0.88)						
5	SWLB	3.52	0.94	−0.31^**^	−0.17^**^	−0.35^**^	−0.49^**^	(0.96)					
6	Well-being	3.35	1.04	−0.14^**^	−0.13^**^	−0.20^**^	−0.31^**^	0.49^**^	–				
7	Gender	1.51	0.50	−0.08	0.05	0.02	−0.06^*^	0.07^*^	0.05	–			
8	Age	46.07	9.82	−0.04	−0.09^**^	−0.12^**^	−0.08^**^	0.04	0.10^*^	−0.03	–		
9	Average weekly work hours	42.92	118.72	0.02	0.04	−0.01	−0.01	0.02	0.01	−0.04	0.02	–	
10	Physical health	3.70	0.91	−0.15^**^	−0.10^**^	−0.27^**^	−0.39^**^	0.35^**^	0.35^**^	0.08^**^	−0.05	0.01	–

**Correlation is significant at the 0.01 level (2-tailed). *Correlation is significant at the 0.05 level (2-tailed). Variables 1–6 are reported as averages across the whole timespan. For gender, 1 = male, 2 = female. Reliabilities (coefficient alphas) appear on the diagonal in parentheses.

### Hypotheses testing

Our dataset consisted of two hierarchically nested levels: observations spanning the seven time points (Level 1 *n* = 1,143) were nested into individuals (Level 2 *n* = 198, with 118 individuals having responded to all seven surveys). We used hierarchical linear modeling (random coefficient modeling) to test our longitudinal model using MLmed (Beta 2), a computational macro for SPSS (v25) (Hayes and Rockwood, 2020; Rockwood and Hayes, in press).

The results of these analyses appear in Table 3. The first column presents frustration as an outcome variable. Hidden work was positively related to frustration at the between-individuals level (*effect size =* 0.08, *se =* 0.04, *p <* 0.04), supporting Hypothesis 1, but not at the within level (across time).

**Table 3:** Results of longitudinal mediation analyses using MLmed.

Predictors / outcome variable	Frustration (mediator)	Stress	SWLB	Burnout	Well-being	Physical health
*Within-effects (across time)*						
Constant	3.06^**^ (0.26)	1.15^**^ (0.37)	5.40^**^ (0.34)	−0.00 (0.45)	4.39^**^ (0.43)	5.40^**^ (0.34)
Hidden work	−0.03 (0.07)	0.19^**^ (0.04)	−0.17^**^ (0.03)	0.22^**^ (0.04)	−0.06^†^ (0.03)	−0.17^**^ (0.03)
Frustration		0.15^**^ (0.02)	−0.02 (0.02)	0.09^**^ (0.02)	−0.01 (0.02)	−0.02 (0.02)
*Between-effects (between individuals)*						
Hidden work	0.08^*^ (0.04)	0.31^**^ (0.05)	−0.21^**^ (0.05)	0.29^**^ (0.06)	−0.07 (0.05)	−0.21^**^ (0.05)
Frustration		0.38^**^ (0.07)	−0.48^**^ (0.07)	0.71^**^ (0.09)	−0.39^**^ (0.08)	−0.48^**^ (0.07)
Age	−0.01^†^ (0.00)	−0.01 (0.01)	−0.00 (0.01)	−0.00 (0.01)	0.01 (0.01)	−0.00 (0.01)
Gender	0.13^†^ (0.08)	0.09 (0.07)	0.07 (0.06)	0.09 (0.08)	−0.02 (0.07)	0.07 (0.06)
Work hours	0.00 (0.00)	−0.00 (0.00)	0.00 (0.00)	−0.00 (0.00)	0.00 (0.00)	0.00 (0.00)
Within indirect effect of hidden work via frustration on outcomes		−0.0044 (LLCI = −0.0247; ULCI = 0.0152)	−0.0005 (LLCI = −0.0025; ULCI = 0.0044)	−0.0028 (LLCI = −0.0165; ULCI = 0.0099)	0.0004 (LLCI = −0.0024; ULCI = 0.0041)	0.0005 (LLCI = −0.0025; ULCI = 0.0044)
Between indirect effect of hidden work via frustration on outcomes		0.03 (LLCI = 0.0001; ULCI = 0.0649)	−0.04 (LLCI = −0.0771; ULCI = −0.0006)	0.03 (LLCI = −0.0001; ULCI = 0.1142)	−0.03 (LLCI = −0.0666; ULCI = −0.0001)	−0.02 (LLCI = −0.0436; ULCI = −0.0001)
Model fit (AIC)		7073.54	6401.15	6978.78	6589.09	6328.98
Model fit (BIC)		7096.46	6432.06	7001.70	6612.01	6351.89

^*^p < 0.05; ^**^p < 0.01; ^†^*p* < 0.10. LLCI, lower-level confidence interval; ULCI, upper-level confidence interval.

Turning to mediation analyses, on the between-individuals level frustration was shown to carry a positive indirect effect of hidden work on stress, with confidence intervals excluding zero (indirect effect *size =* 0.03, *LLCI =* 0.0001, *ULCI =* 0.0649), supporting Hypothesis 2a. The indirect effect of hidden work on SWLB via frustration was also significant at the between level (indirect effect *size =* −0.04, *LLCI =* 0.0771, *ULCI =* 0.0006), giving support for Hypothesis 2b. Moreover, the indirect effect of hidden work on burnout via frustration was also significant at the between level (indirect effect *size =* 0.03, *LLCI =* 0.0001, *ULCI =* 0.1142), supporting Hypothesis 2c. Finally, the indirect effect of hidden work on subjective well-being via frustration was significant at the between level as well (indirect effect *size =* −0.03, *LLCI =* −0.0666, *ULCI =* −0.0001), lending support for Hypothesis 2d. Supporting Hypothesis 2e, the indirect effect of hidden work on physical health via frustration, was also significant at the between level (indirect effect *size =* −0.02, *LLCI =* −0.0436, *ULCI =* −0.0001). No indirect effect on the within level (across time) was significant, indicating that noteworthy variation in the variables driving the relationships we studied occurs between individuals, not within individuals, and changes over time.^2^

## Discussion

Based on the organizational model of frustration, we proposed and tested the relationships between hidden work, frustration, and multiple layers of health. The results of our longitudinal (seven-repetition) study, conducted during the first and second periods of the pandemic showed that COVID-19-situation-induced hidden work invoked emotional responses of frustration that then influenced outcomes consisting of multiple layers of occupational health—positively affecting stress and burnout, and negatively influencing SWLB, subjective well-being, and physical health. In summary, our findings suggest that employees who adhere to the “show must go on” philosophy in these challenging times, as reflected in higher levels of hidden work, are thereby undermining their health on multiple levels. These findings could change the attitudes of employees, employers and other key stakeholders to recognize hidden work as an important source of occupational health problems.

### Theoretical contributions

This study seeks to contribute to theory in three distinct, yet interrelated ways. First, we contribute to the occupational health literature by theoretically developing and empirically testing the concept of multiple layers of occupational health as an outcome of the COVID-19-induced work changes and emotional responses. Prior research on occupational health outcomes is scattered across different conceptualizations and considerations of this phenomena, which often stem from different disciplines like organizational behavior/psychology, and occupational health and safety. Previous attempts to reconciliate and integrate off outcomes that cover multiple dimensions of health vis-à-vis work-related occurrences still largely focus on a single aspect such as psychosocial health (Petterson and Arnetz, 1998), or focus predominantly on a specific level or output beneficiary of health considerations, such as an individual or an organization (Campbell Quick et al., 2007).

Advancing the majority of occupational health research, which considers individual dimensions of this concept (Cooper and Cartwright, 1994; Schaufeli and Greenglass, 2001; Mikkelsen et al., 2005), we offer an integrative, multi-layered concept of occupational health that encompasses physical, psychological and psycho-social aspects. In so doing, we followed the WHO’s four-layered healthy workplace model (Burton and WHO, 2010). This holds important implications for the field of occupational health across different disciplines that concentrate on these phenomena. Such a multi-layered conceptualization provides a more comprehensive view of health-related phenomena that can be applied while examining its determinants in setting up a workplace that considers the human factor and places individuals at the center of the investigation, rather than merely organizationally-relevant outcomes. While most recent studies examined the pandemic’s impact on a specific group of employees (e.g., healthcare workers, remote workers) during the pandemic’s first wave, we extend the growing body of literature on occupational health during COVID-19 by examining the pandemic’s influence on a nationally representative quota sample of employees working in a broad of industries and conducting a longitudinal study during the first and second waves of the pandemic.

Second, we view the organizational model of work frustration as a backbone for identifying different sources of frustration (Spector, 1978) to advance subsequent research on the matter by examining it in the COVID-19 pandemic context. We complement existing research on frustration at work by proposing that hidden work occurs because of environmental sources of frustration due to an uncertain situation, thereby proposing a dual-layered model of sources of frustration. On one hand, frustration has been related to work occurrences, which is a much more prevalent line of investigation in the extant literature (*cf.*, Fox and Spector, 1999; Harvey et al., 2010; Vander Elst et al., 2012). Despite this rich body of literature concerned with the relationships of frustration with important negative work-related outcomes, frustration as a form of psychological strain has received very little empirical attention (Whinghter et al., 2008).

In accordance with frustration theory (Maier, 1949), stressors (e.g., quantitative workload) act as frustrating agents (Whinghter et al., 2008). Nevertheless, previous literature has not directly investigated how individual work-related characteristics might influence the development of affective outcomes like frustration over time. Our longitudinal study provides causal evidence for the effects of workload/overload (hidden work) on the development of the emotional response of frustration and subsequent outcomes, which not only empirically but also theoretically relevant given that it provides a rigorous test of the directionality of the effects proposed in the literature.

On the other hand, one overlooked area is sources of frustration arising from the broader situation, in our case, the uncertainty and anxiety resulting from the general COVID-19-induced atmosphere in societies and organizations. We focus directly on the role of context above and beyond the organization, complementing research on organizational climate (Chang et al., 2018), evaluative context, and direct supervision (Morbée et al., 2020), or bureaucracy (Lewandowski, 2003) as sources of frustration. We apply a dual-layered conceptualization derived from the original model of work frustration (Spector, 1978) to enrich the nomological net of tested outcomes of the frustration model with regard to the aforementioned multiple dimensions of occupational health. Although frustration refers to an individual feeling, the majority of studies examined the influence of frustration on organizationally-relevant outcomes such as job performance, work engagement, absenteeism, and organizational and interpersonal aggression (e.g., Fox and Spector, 1999), thereby neglecting the potential negative individual-level consequences. We go beyond existing studies by making a theoretical and empirical attempt to establish a critical link between frustration and health outcomes (i.e., individual-level consequences) during the COVID-19 (and similar/potential) crises.

Third, we add to the research on hidden work and workload/overload in general. Prior research indicated that hidden work brings certain negative outcomes (Barley et al., 2011; Kost et al., 2023), but also found positive consequences for workers and organizations like task performance (Fenner and Renn, 2004, 2010). However, this research has not specified particular emotional responses resulting from hidden work, nor has it explained the mechanisms through which they affect outcomes. We add to understanding of its effects on occupational health-related outcomes, thereby complementing existing research on hidden work that shows it contributes to a less favorable work–life balance and perceptions of work overload (Barley et al., 2011; Kost et al., 2023), as well as lowering perceptions of well-being (Eichberger et al., 2021). We advance this stream of research by conceptualizing and testing a mechanism of how hidden work relates to multiple layers of occupational health outcomes by specifically identifying hidden work as a source of frustration, thereby also addressing calls for further examination of the potential negative effects of workload and overload generally (Győrffy et al., 2016). As digitalization increases and thus also the consequent potential for hidden work outside working hours, these findings are extremely relevant also beyond the current pandemic situation.

### Practical implications

The presented findings also hold important practical implications for setting up work-related policies regarding workload, especially in the light of urgent situations. To survive the COVID-19 crisis, or crises of a similar nature, organizations often (intentionally or unintentionally) force employees to make extraordinary efforts to keep the business running. In order to hold on to their job and the financial stability that comes with it, employees are often willing to follow the “show must go on” philosophy and work long hours even in their personal time without receiving any additional compensation. At first glance, while this seems to be a rational strategy, our findings suggest that in the long run it can negatively influence employees’ mental and physical health, in turn seriously jeopardizing the continuity of business processes. Organizations should therefore do more to keep after-hours work to a minimum (e.g., by implementing a “no after-hours” or “limited timeframe email” policy). For example, organizations can ensure that formal expectations of employees’ availability at all times and places remain low (Piszczek, 2017), and encourage them to take time off from work. It is important to make it clear that this will not cause them to miss work-related opportunities or fall behind in their work activities.

The issues with hidden work are especially prevalent when it comes to remote work arrangements, even now that the effects of the COVID-19 pandemic are fading. The opportunity for individuals to work from home and the resulting autonomy can encourage hidden work and negative health-related outcomes. Still, reducing the opportunity for employees to work from home has also been shown to bring negative consequences (Raghuram et al., 2019). Such a situation therefore induces a paradox concerning how much flexibility is actually enabled. This paradox may be alleviated by promoting explicit separation norms regarding the separation of work from personal life (Kossek et al., 2006; Kost et al., 2023). Due to the potential negative impact of hidden work via frustration on employees’ health on multiple levels, protective mechanisms should be introduced to systematically prevent the occurrence of hidden work This would constitute an important step towards creating a healthy workplace in times of crisis and in the light of the potential negative effects of digitalization, and hence in protecting employees’ health. In addition to formalized norms, managers and leaders in particular must act as role models for their employees by not answering emails and not being present on other work-related channels.

## Limitations

Like any study, ours is not without limitations. While our longitudinal seven-time-points investigation has great advantages in terms of causal statements, a possible limitation of the research design is that we refer exclusively to self-reports. However, earlier research has reported that by focusing on these perceptions, rather than the objective reality that would be expected to lead to experienced frustration and associated affective and behavioral responses, self-report measures may more adequately capture critical features of the situation than more objective, non-incumbent measures would (Fox and Spector, 1999). Wherever the research aim is to understand how people view, feel about, and respond to their jobs, self-report methodology may be most useful (Howard, 1994; Schmitt, 1994; Spector, 1994). Still, such research could be supplemented by the inclusion of additional objective measures of health-related outcomes, such as sick leave days, objective investment by organizations in workplace health and safety initiatives etc. In terms of the measurement instruments, we only used single-item measures for several constructs (i.e., for frustration, stress, subjective well-being, physical health). While prior research has demonstrated the validity of such single-item measures (*cf.*, Elo et al., 2003), and this approach may prove particularly useful in longitudinal research to avoid overwhelming respondents with research instruments that are too long, enabling them to maintain concentration on the content while responding (Lucas and Donnellan, 2012; Fisher et al., 2016). Future research could further improve the validity of our study by employing longer and multi-dimensional scales.

Another limitation of our research can be seen in the fact that we did not control for different flexibility arrangements among employees prior to the occurrence of the COVID-19 pandemic and other contractual agreements concerning overtime. Various types of flexible work arrangements may increase the amount of hidden work employees perform (Chung and van der Horst, 2020). This can potentially affect the relationship between hidden work and its outcomes and, although we measured these in the second period of our data collection (time points 5–7) and it was evidenced that did not play a role there, a comprehensive investigation of these factors is nonetheless warranted. If flexible working arrangements are introduced to improve employee performance, this may be a factor in the effects of hidden work on various outcomes.

Further, segmentation norms may also affect the extent to which employees engage in hidden work (Derks et al., 2015). Future research should consider these factors while additionally explore the effects of hidden work on proximal and distal work outcomes, potentially look at the interplay among occupational health outcomes to test the proposed sequence in which they occur (with respect to temporality and the severity of their impact), and continue to focus on outcomes beyond occupational health-related ones, such as multiple layers of performance (task, contextual, creativity etc.).

## Data availability statement

The raw data supporting the conclusions of this article will be made available by the authors, without undue reservation.

## Ethics statement

Ethical approval was not required for the studies involving humans because at our institution, we do not require ethics committee approval for behavioral perception studies that are noninvasive, fully anonymized, and confidential. Therefore, when conducting our non-interventional study (i.e., survey), we fully informed all participants that their anonymity was guaranteed, why the study was being conducted, and how the data would be used. The studies were conducted in accordance with the local legislation and institutional requirements. The participants provided their written informed consent to participate in this study.

## Author contributions

MČ and DA designed the model and computational framework, conceived the study, and drafted the manuscript. MČ performed the calculations. All authors discussed the results, commented on the manuscript, and worked on the final version of the manuscript.

## Footnotes

^1^Also labeled as teleworking, virtual working, distance working, telecommuting, or working from home. It is an important aspect of the changed nature of work during the pandemic and, as evidenced by our research, the majority (62%) of the respondents did work from home during it. However, the model presented and tested in our study is not only applicable to remote work.

^2^The results of testing the hypotheses also hold for each period separately, and for the second period when controlling for the dummy of *working remotely* or not.
